# Building on momentum from the global campaigns: an exploration of factors that influenced prioritization of stillbirth prevention at the national level in Uganda

**DOI:** 10.1186/s12992-021-00724-1

**Published:** 2021-06-26

**Authors:** Eric Ssegujja, Michelle Andipatin

**Affiliations:** 1 Department of Health Policy Planning and Management, Makerere University School of Public Health, Kampala, Uganda; 2 School of Public Health, University of the Western Cape, Cape Town, South Africa; 3 Department of Psychology, University of the Western Cape, Cape Town, South Africa

**Keywords:** Stillbirth, Global campaigns, National prioritization, Norm promotion, Policy Community, Maternal and Child Health

## Abstract

**Background:**

Of the close to 2.6 million stillbirths that happen annually, most are from low-income countries where until recently policies rarely paid special attention to addressing them. The global campaigns that followed called on countries to implement strategies addressing stillbirths and the adoption of recommendations varied according to contexts. This study explored factors that influenced the prioritization of stillbirth reduction in Uganda.

**Methods:**

The study employed an exploratory qualitative design adopting Shiffman’s framework for political prioritization. Data collection methods included a document review and key informants’ interviews with a purposively selected sample of 20 participants from the policy community. Atlas. Ti software was used for data management while thematic analysis was conducted to analyze the findings.

**Findings:**

Political prioritization of stillbirth interventions gained momentum following norm promotion from the global campaigns which peaked during the 2011 Lancet stillbirth series. This was followed by funding and technical support of various projects in Uganda. A combination of domestic advocacy factors such as a cohesive policy community converging around the Maternal and Child Health cluster accelerated the process by vetting the evidence and refining recommendations to support the adoption of the policy. The government’s health systems strengthening aspirations and integration of interventions to address stillbirths within the overall Maternal and Child Health programming resonated well.

**Conclusions:**

The transnational influence played a key role during the initial stages of raising attention to the problem and provision of technical and financial support. The success and subsequent processes, however, relied heavily on domestic advocacy and the national political environment, and the cohesive policy community.

**Supplementary Information:**

The online version contains supplementary material available at 10.1186/s12992-021-00724-1.

## Introduction

Globally up to 2.6 million stillbirths occurred in 2015 with most of the cases from Low and Middle-Income Countries (LMIC), in rural areas, and during the intrapartum period [1–3]. Campaigns to draw attention to the problem called for particular attention especially in the policy arena to respond to prenatal health and the survival continuum of care [4]. Earlier responses included estimation of the burden [3] although this was constrained by inadequate data due to inadequate data captured in the vital statistics [5]. This was evident while compiling the countdown to the Millennium Development Goals (MDG) reports which despite its burden, was initially not one of the indicators for tracking [6]. As global momentum grew punctuated by “Call to action”, “ENAP” and “Ending preventable stillbirths” so were national-level efforts to translate campaign recommendations into service delivery [1, 7–9].

Adaptation of global recommendations into national and subnational level service delivery involves negotiating blurred lines between interventions to address maternal and neonatal mortality[10]. As such, the potential to save mothers and neonates led to calls for the strengthening of health systems for a triple return on investment[11]. However, despite this potential, it is not clear why stillbirths did not receive the same rates of reduction as maternal and child health[12]. Maternal and Child Health (MCH) Interventions include the promotion of early antenatal care attendance and completion of the recommended antenatal care visits as well as the promotion of facility delivery under a skilled attendant. For this study, we restrict the discussion of interventions to address stillbirths that were promoted through the global campaigns whose momentum peaked with the 2011 Lancet stillbirth series: call to action. Stillbirth is understood as fetal death after twenty-eight weeks of gestation[13]. The campaign postulated that improving care around delivery through offering Emergency Obstetric Care services have the greatest effect with syphilis treatment having a moderate effect while advanced Antenatal Care (ANC) would have the least effect. Delivering such services at Universal coverage (99 %) was estimated to lead up to 45 % of third-trimester stillbirths averted on top of 54 % maternal deaths and 43 % neonatal deaths averted per year[9, 10].

In Sub-Sahara African region and Uganda in particular, delivering interventions to address stillbirths include improving the quality of services during the intrapartum period, antenatal care, and along the continuum of care for women’s health. Available evidence suggests that interventions during the intrapartum period have the potential of addressing most risk factors once identified early with relevant remedies provided. [10]. These include facility delivery under a skilled birth attendant and delivery of emergency obstetric care services. In Uganda, several interventions have been implemented in this direction such as operationalization of Health Centre (HC)IVs to deliver comprehensive, Emergency Obstetric care with HCIII offering basic emergency obstetric care and HCIIs delivering outpatient maternal health services among others[14]. Interventions implemented during antenatal care include detection and management of maternal disorders and fetal complications while interventions along the continuum of care for women’s health include; folic acid fortification, sleeping under insecticide-treated bed nets, malaria prevention using the intermittent preventive treatment, and syphilis detection and management[9]. Some interventions are currently integrated within the routine standard of maternal health care services with referrals recommended at service provision levels unable to offer the required services [14].

A demand-side gradient to the causes and response to stillbirths is also evident. The high rates of teenage pregnancies in Uganda mean increased risks of stillbirth[15]. The distance to health facilities is still a major barrier to health services access where the average distance is within five kilometers [14]. Inadequate knowledge about stillbirth and measures to mitigate the risk factors [16] contributes to the poor health-seeking behaviors for maternal health services as reflected in suboptimal completion of recommended visits and health facility deliveries [17]. The resultant effect is the missed opportunity for women on this vital care crucial for identifying and responding to stillbirth risk factors. The negative cultural dynamics surrounding maternal healthcare seeking decision making coupled with stillbirth taboos impede support seeking due to stigma attached to the mother and her entire family[16]. At the health system level, postnatal care services offered to mothers after a stillbirth is still inadequate[18].

More remains to be seen on how national-level governments embraced global stillbirth campaign recommendations and translated them into service delivery. Evidence from similar efforts within maternal and child health demonstrates mixed levels of adaptation [19, 20]. In some of the cases, countries were quick to embrace the recommendations and results were visible on some key indicators while it takes time for others[20]. In Malawi and Mali, for example, initiatives to promote neonatal survival led to the adoption of effective interventions[21]. Slow adaptation of global campaigns led to sub-optimal progress in addressing maternal mortality reduction in Nigeria[20].

The global response towards the stillbirth burden offers a unique opportunity to understand the influence of global campaigns on national-level policy processes and political prioritization. According to Shiffman (2007), political priority is present when leaders publicly and privately express concern and support for an issue, the government through its legislative function enacts policies and guidelines that embrace strategies to address the problem; and the government allocates and releases funds commensurate to the problem[20]. In Uganda, the accelerated political prioritization was reflected in efforts to implement interventions geared towards stillbirth reduction. This followed global strategies aimed at reduction in stillbirth rates, the campaigns prioritized regions with the highest contribution to the burden which included sub-Saharan Africa and Asia[13]. The initiative called upon countries to strengthen health systems, implement proven high-impact low-cost interventions during the antenatal and intrapartum periods along the continuum of care for women and children[10]. We noted recommendations for the integration of targeted interventions within women and children’s health programs from the campaigns [22]. Country-level data on the extent to which these recommendations were implemented remains minimal since each country adopted varying strategies according to their health systems capacities [10].

The global campaigns played a key role in drawing attention to stillbirths as a neglected public health problem [11]. The campaign was in part accelerated by its adoption within the United Nations (UN) systems thereby committing member countries to devise and implement strategies to reduce the burden at the national level [8]. In 2014, the “Every Newborn Action Plan” (ENAP) set a stillbirth reduction target of 12/1000 or less by 2030. This reflected commitment towards the set targets and implementation of strategies at the country level has varied based on contexts as witnessed elsewhere from country-level experiences [20]. There remains a paucity of information on the extent of prioritization and the underlying factors that may have influenced prioritization of stillbirth reduction at the national level. We were not able to identify any previous study that conducted an in-depth investigation of how global stillbirth campaigns influenced prioritization on the national health agenda in regions that were identified as contributing to the highest global burden. The main objective of this paper was to explore and understand the factors that influenced the prioritization of stillbirth reduction on the national health agenda in Uganda.

## Methods

### Study design

The study adopted an exploratory qualitative design as part of a larger mixed-methods study. To capture national-level factors that influenced prioritization of stillbirth reduction, a national-level qualitative study employing a document review and key informants’ interviews with respondents knowledgeable about national-level maternal and child health policy process drawn from established policy networks was conducted.

### Framework

We adopted the Shiffman framework for analyzing political prioritization[20]. It analyses nine factors grouped into three dimensions that include transnational influence, domestic advocacy, and national political environment. It is described in detail in the Table 1 below;

**Table 1:** Shiffman’s Framework for analyzing Political Priority

Category	Factor	Description
Transnational influence	Norm promotion	Efforts by international agencies and actors to establish global norms
	Resource provision	Provision of financial and technical support from international agencies to address the problem.
Domestic advocacy	Policy Community cohesion	The degree to which national-level promoters coalesced as a political force to push the government to act.
	Political entrepreneurship	The presence of respectable and capable national champions willing to promote the cause.
	Credible indicators	The availability and strategic deployment of evidence to demonstrate the presence of the problem.
	Focusing events	The organization of forums to generate national attention to the cause.
	Clear policy alternatives	The availability of a clear policy alternative to demonstrate to political leaders that the problem is surmountable.
National Political environment	Political transition	Political changes that positively or adversely that affects prospects for promotion
	Competing health priorities	Priority for other health causes that divert policymakers’ attention away from the problem

### Study sample

The sample comprised of purposively selected individuals from the national level maternal and child health policy communities. At the design stage, the inclusion criteria were set to interview only those respondents that had spent at least two years in their positions. These key informants were pre-identified as eligible for interview. This was the same criteria applied to respondents that were snowballed after study commencement from leads provided during the interviews. A list of potential respondents with their contacts was generated following consultations with contacts familiar with national-level maternal health policy processes. Additional respondents were selected based on leads from the Ministry of Health (MoH) depending on the contribution of such individuals to the policy processes. They were drawn from the Ministry of Health, professional associations, implementing partners, academia, and the private sector.

 Respondents were approached through telephone calls from which the objectives of the study were explained and they were asked if they were willing to participate. Indication of willingness to participate was followed with arrangements for a day, time, and respondent’s convenient secure, and convenient place when the interview would be conducted. A total of 20 key informants were interviewed with only three potential respondents who had indicated willingness to participate were not interviewed after failing to schedule an interview on the third call back. The common reason given by two of them was the busy schedule as this was the time of finalizing the Health sector budget for the subsequent financial year beginning July 1st while the third potential respondent could not find time within the data collection period. For the document review, a process tracking method of key events guided the sampling of key documents used in this review. They included; discussion papers, reviews, original studies, editorials, commentaries, web articles, government policy documents, and guidelines, as well as reports from government and other organizations.

### Data collection

The data collection process deployed two main methods including key informant interviews and document review. Key informant interviews were conducted by the first author (ES) assisted by two female graduate-level research assistants. The key informant interviews were conducted between March and June 2019 primarily at the respondent’s places of work or any other preferred convenient location. Interviews were conducted face to face with audio recording with field notes taken during the interview lasting between 45 min to one hour depending on the point at which saturation was attained. An interview guide was specifically developed for this study by the first author and reviewed by the last author. It contained open-ended questions and probes to stimulate discussion which were later harmonized and organized following the factors reflected in the applied Shiffman’s theoretical framework[20]. A maximum of two callbacks was made in case the first appointment did not materialize after which another respondent would be identified. Field notes were taken during the interview process and at the end of each field day during the debrief meetings. Overall, 17/20 were females and drawn from Ministry of Health [5], Non-Governmental Organizations (NGOs) [4], Professional associations [6], Private not for profit health facilities [2], academia/researchers [2], and from private for-profit [1]. However, the respondents’ places of work were not mutually exclusive as some doubled as providing varying services in the categories used in this study.

The document review involved a search strategy following the process tracing technique [20, 23] guided by key milestones identified in the earlier literature review. It followed global health initiatives to draw attention to the burden of stillbirth and efforts towards reduction as highlighted in global health databases and journals. The second stage included searching through grey literature. This was followed by a search on Google scholar in line with grey literature search approaches used elsewhere[24]. Key organizations involved in stillbirth advocacy were targeted via some of the grey literature. [25]. Lastly, a backward and forward search through reference lists of included documents was done. The document search and analysis[26] was done by the first author with a second review done by the last author (MA) who was the supervisor. The review sought to document the global processes that culminated into campaigns that led to the translation of emerging ideas and frames into national-level prioritization of interventions to address stillbirths.

### Data analysis

All interviews were conducted in English and hence the audio recordings were transcribed verbatim by two research assistants who participated in the data collection. The first author read through each of the transcripts to ensure all the information was captured. Data were analyzed using Atlas. ti a qualitative data management software package[27]. The process involved entering transcripts into the software where a codebook following the Shiffman theoretical framework was developed by the first author and used for coding. Chunks of text relating to a particular code were highlighted and attached to specific codes. Thereafter query reports were run for each of the codes and a manual pile sorting exercise was conducted to identify underlying meanings which led to the grouping of texts with similar meanings under different subthemes within the main framework construct. Finally, we employed the Consolidated Criteria for Reporting Qualitative Results (COREQ)[28] to guide the reporting of the qualitative data where selected typical quotes representing the subtheme were used to support the presentation of the results. To control for bias, analysis of data from the two data collection sources was done concurrently with a back-and-forth triangulation.

### Methodology integration

The research question and the study design were informed partly by the literature review the result of which informed the sub-study investigating national level prioritization of stillbirth reduction into programs and policies. The document review informed the process tracing exercise[23] and was confirmed through key informant recollection of the process and highlighted key events. In the first section of the results, the timelines reflecting international and local events leading to the prioritization of stillbirths were re-constructed. This informed the development of the interview guide used during data collection among national-level key informants whose qualitative results are presented in the subsequent sections. They correspond to the categories highlighted in Shiffman’s framework that include; transnational influence, domestic advocacy, and national policy environment[20]. A complete range of integration of the document review and qualitative results is reflected in the results section where both findings augment each other in explaining the main themes of the study and in the discussion section.

## Results

To understand stillbirth prioritization in Uganda, reference is first made to global events. Table 2 shows that stillbirths did not receive much global recognition before 2005. Earlier national-level efforts were mostly led by bereaved parents organizing themselves to bring the issue of stillbirths to the fore[29]. Specifically, International Stillbirth Alliance (ISA) started in the USA in 2003 by three mothers to stillborn babies aimed to push for improvements in bereavement care, prevention research, and clinical care which has grown into a global movement[30]. The publication of the count-down reports that reflected it as a missing maternal and child health indicator amplified these efforts [31]. Global momentum to draw attention to this omission was building alongside preterm birth and neonatal health and during 2009 a prematurity and stillbirth conference was held where participants designed a roadmap to address the issue[32]. Subsequent initiatives led to the publication of the Lancet stillbirth series in 2011[2] and its inclusion in the countdown report to raise global visibility and call to action[33]. National efforts to prioritize stillbirth reduction were not new to the health systems strategies but rather only received a boost from these global campaigns. With time, they were reflected in the Annual Health Sector performance reports with some interventions to reduce stillbirths as part of program components in maternal and newborn projects. Later, stillbirth reduction strategies were included in the national guidelines.

**Table 2:** Global key events and timelines

Year	Key event	Link to global campaign theme	Relevance to stillbirth response
2003	Foundation of the International Stillbirth Alliance in the USA	Actions against stigma. Research. Improved care. Advocacy	Combine healthcare professional knowledge and passion for families to advance stillbirth prevention research, medical/clinical care, and bereavement services.
2005	First MDG report of the countdown to 2015 with stillbirth not reflected as an indicator	Establish burden and disparities	Absence of stillbirth as an indicator for maternal and neonatal health outcomes later informed WHO’s decision to include it as a quality of care indicator.
2009	Seattle conference convened by Global Alliance for Prevention of Prematurity and Stillbirth (GAPPS)	Evidence of cost effective interventions Global and country targets for stillbirth reduction	Drawing global attention to pre-term and stillbirth which had shown less progress compared to maternal and child mortality.
2010	Countdown to 2015 decade report (2000–2010): taking stock of maternal, newborn and child survival	Establish burden and disparities Integrated prevention	• Considering stillbirth as a vital indicator for maternal and child health outcomes. • Provision of evidence on scale of stillbirth and amplifying the global burden. • Raising visibility • Promote prioritization of intervention to address the burden.
2010	Launch of the UN Secretary general’s Global strategy “Every woman every Child”	Integrated prevention	Highlights key areas for urgent attention to enhance financing, policy and service delivery with a newborn survival component which included addressing stillbirth
2011	Lancet stillbirth series: Call to action	Evidence of cost effective intervention. Progress monitoring. Integrated prevention. Investigate causes. Establish burden and disparities. Research. Action against stigma. Advocacy.	Reviewed status of stillbirth and advocacy to get stillbirth out of the shadow with a call to all stakeholders to take action geared towards reduction.
2011	Launch of saving Lives at Birth	Integrated prevention. Evidence of cost effective interventions.	Increased funding from global health stakeholders for maternal and child health interventions with neonatal component and specifically targeting combating preventable stillbirth
2012	Rollout of Saving Mothers Giving Lives (SMGL)	Integrated prevention. Evidence of cost effective interventions. Progress monitoring Actions against stigma	• Enhance existing district maternal and child health services to strengthen evidence-based interventions through a three delays model. • Reduce pregnancy and childbirth related deaths including stillbirth and primarily focusing on the critical period of labor, delivery, and 48 h postpartum when most maternal and newborn deaths happen. • Harnessing of the public-private partnership.
2013	First Global Conference on newborn survival held in April 2013 in Johannesburg South Africa.	Evidence of cost effective interventions. Integrated prevention Progress monitoring. Establish burden and disparities. Global and country targets for prevention.	• First Global conference for newborn summit aimed at accelerating scale-up of high impact interventions to address leading causes of newborn mortality. • Review progress to tackling preventable newborn deaths and call to action for urgency to address the problem. • Develop ENAP in support of global strategy for Women’s and children’s health, Every woman Every Child movement and build recommendations for UN commission on Life saving commodities, A promise Renewed to child survival and Family Planning 2020 objectives.
2014	Every newborn action plan (ENAP) in support of earlier Every Woman Every Child.	Integrated prevention. Evidence of cost effective interventions Global and country targets for prevention.	• Advances the objectives of the global strategy for women and children by focusing on quality of care at birth with special attention to newborn health and stillbirth as unfinished agenda from the MDGs • Sets global and national targets for preventable stillbirth reduction and milestones for quality of care.
2015	World Bank Business plan-GFF in support of Every Woman Every Child	Integrated prevention. Evidence-based cost effective interventions.	Operationalizes the UN Secretary general’s global strategy objective of innovative approaches to financing for health in response to the funding gap to address the RMNCAH unfinished agenda post MDG including reduction of stillbirth
2016	Lancet series; ending preventable stillbirths	Progress monitoring. Evidence of cost effective intervention. Integrated prevention. Establish burden and disparities. Research Action against stigma. Advocacy.	Global advocacy and call to action to address preventable stillbirth
2016	ISA five year strategic plan	Progress monitoring. Evidence of cost effective interventions. Integrated prevention. Action against stigma. Advocacy	Through the strategic plan led to establishment of technical working group strategy to pursue objectives of the five year duration while coordinating international response. Establishment of technical working groups for global coordination of efforts
2016	BMC stillbirth series (care after stillbirth)	Actions against stigma. Evidence of cost effective intervention Integrated prevention	Burden of stillbirth, impact on families and calls for action to address social determinants of health which are the underlying causes. Calls to link research to interventions to address the causes
2020	UNICEF/WHO stillbirth epidemiology report	Establish burden and disparities. Progress monitoring.	Global estimates of stillbirth burden.

### Transnational influence

#### Norm promotion

A key factor for transnational influence was the promotion of norms critical for addressing a public health challenge. Initially excluded as one of the indicators for tracking under the MDGs, stillbirth came to the fore while preparing the report for the countdown to the MDG targets in 2015[33]. The report was intended to act as an accountability measure to keep the MDG pace while recognizing achievements[12] wherein the 2010 countdown report reflected stillbirth as one of the indicators for tracking. The global estimate of stillbirth was approximately 18.9 per 1000 total births in 2009 translating to 2.64 million stillbirths worldwide [13]. Global campaigns held strong views that the burden of stillbirth was unacceptably high, receiving less attention from the health systems, negative cultural practices characterized by secret burial practices[11, 16] and yet many of the cases were largely preventable [33]. A call to policymakers and health systems managers in all countries was to pay attention to this problem by implementing proven low-cost interventions to address the same while global actors were called upon to increase global visibility and dedicate resources especially in regions with the highest-burden [10]. A Global Alliance to Prevent Prematurity and Stillbirth (GAPPS) conference held in May 2009 in Seattle [32] set an objective of developing a roadmap for global action. At the same time, the International Stillbirth Alliance (ISA) earlier started in 2003 by bereaved parents in the USA was expanding with a global influence. They drew attention to the problem while forming alliances with other national-level associations with shared objectives [29].

The 2011 Lancet stillbirth series was a call to action which adopted a mix of advocacy and hard data to promote normative consensus and was estimated to have reached 1 billion people in coverage[4, 34]. It drew attention to the invisibility of stillbirths from the global statistics and at the family level with secret bereavement rituals [11] while highlighting the potential of available low-cost interventions to address neonatal mortality and stillbirth [3]. The 2011 Lancet series was an effective tool for global advocacy[9] which called for the prioritization of stillbirth reduction by at least half of the 2009 baseline of 18.9 per 1000 total births by 2020, increase investment in stillbirth research while improving data systems[7]. The UN embraced the norm by endorsing the ENAP during the World Health Assembly in 2014, which operationalized the earlier United Nations (UN) Secretary General’s Global Strategy “Every Woman Every Child” through the World Health Assembly (WHA) resolution 67. Member countries committed to the strategy with explicit stillbirth reduction targets of 12/1000 by 2030 10/1000 total birth by 2035 especially for high burden countries [8]. Stillbirths were reflected as an indicator within the Maternal Newborn and Child Health [35]. Reflection of stillbirth as a vital indicator with deliberate actions to address the burden became a norm for the UN member countries to adopt. Uganda was one of the countdown priority countries and ENAP countries reporting country progress. ENAP targets were reflected in the Investment Case for the Sharpened Plan[36], the Health Sector Development Plan (HSDP) [14]. Besides, it was reported annually as an efficiency and quality of care indicator in the Annual Health Sector Performance Report (APHSR) [17]. Details are reflected in Table 2 below;

#### Resource provision

Technical and financial resources are critical for the political prioritization of public health problems. For stillbirths, political prioritization was enhanced by the increased funding for maternal and child health with interventions having a neonatal health component. Uganda is a recipient of numerous grants supporting projects that have generated evidence to address stillbirths. As one of the pilot countries for the Saving Mothers Giving Life project [37, 38]that piloted the use of the BABIES Matrix, the evidence-informed revisions to the national Maternal and Perinatal Death Surveillance and Responses (MPDSR) guidelines[39]. It streamlined the audit of perinatal death and helped in improving the classification of stillbirth as well as informing appropriate interventions. This was re-echoed by a national-level key informant that worked closely in generating and disseminating this evidence below;

* Well [the] Ministry is spearheading along with implementing development partners to see that they tighten up on guidelines first of all; …. and there is also an aspect on quality improvement that the team focuses on and maybe I will just cite one example that we fronted to the Ministry of Health, it is called The Babies Matrix which is a quality improvement tool. It is a very simple tool for one to use both at the facility and even at the community level that focuses just on birth weight and age at death. So if you are able to collect that data you are able to determine the different categories of newborn deaths both pre-discharged and also those who died at the intrapartum; … and to know the various interventions to target. So those are the things that were fronted by the project and we believe through the continued technical working groups that are happening at the Ministry it is something that would be taken to scale.****(KII_NLI019)***.

The World Bank’s support through the Global Financing Facility has one of its aims to reduce 21 million stillbirths in high burden countries by 2030[40]. Through this support, Uganda is implementing an integrated health systems approach that has fast-tracked implementation of interventions to address stillbirths[17] in line with the first MCH conference-2015 statement calling for the implementation of scalable programs beyond pilots [41]. Other components under this support include improvements in data capturing through support for a community arm and civic registration systems, operationalize the ENAP strategic objective to count every newborn through investing in birth and death registration[42]. A respondent thus noted;

*we have accessed a loan (WB) whereby it has 3 components and one is system strengthening and the other is result-based financing at least to finance health care delivery through the system and the 3rd component is through National Identification and Registration Authority (NIRA). We have birth and death registration. So Ministry of Health is working together with NIRA to develop the tools, to build a system that can capture the data and deaths and also the aspects on macerated deaths, some few reasons why that death happened for quality so that we have a system of notifying the maternal deaths and the perinatal deaths and also we shall go into another arm of notification which is the verbal autopsy. The biggest deaths of mothers are in communities and this system(DHIS2) cannot capture that but now with NIRA, we shall have a community arm through maybe the VHTs, maybe it could be a community system to capture them and they are notified to the districts to the NIRA office and then we capture that. It will improve on our notification and registration****(KII_NLI007)***.

International organizations and funders also contributed resources specifically towards fast-tracking the policy-making processes to streamline policies responsive to stillbirths as echoed by a respondent.

*Currently, UNICEF is sponsoring the Newborn Steering Committee meetings to see that the policies on newborns are going ahead. They work with UNFPA that is really improving maternal mortality rate. They are working with different [partners]; like Save the Children, USAID to see that some of these policies are implemented. I think they are trying their level best, and they are also working with different associations like AOGU, UPA and WHO as well.****(KII_NLI016)***

### Domestic advocacy

#### Policy community cohesion

National efforts to enhance stillbirths as a political priority are partly attributed to a cohesive policy community converging around the Maternal and Child Health-Technical Working Group (MCH-TWG) known as the MCH cluster. Its diverse composition included researchers, professional associations, practitioners, implementing partners, policy implementers, and policy makers among others. The group sifts through the evidence for policy consideration[39] and its members have previously supported moves for resource mobilization [43]. The level of organization and proximity to decision-making worked in favor of promoting stillbirth prioritization. Commenting about the work of this team, a respondent thus noted;

* we have what we call the RMNCH [cluster]; the technical working group which meets every month and now there is a bigger forum which brings on board other multi-sector practitioners who meet on a quarterly basis. So these two I think have added a lot of value because when we meet we share experiences and we try to identify the bottlenecks and solutions. I think that has helped and of course there is also the Health Assembly which is held once a year that brings together even practitioners from the District. So all these are forums that have harnessed the synergies of civil society, private practitioners to come together and find ways of improving this, but the Assembly also gives an opportunity to citizens to speak and say what they think.****(KII_NLI013)***.

The ability to mobilize members and vet on issues where evidence is synthesized before recommending policy actions was mentioned as one of its strengths. A respondent recalled an incident where this technical working group vetoed against a guideline which was being pushed without their involvement and other stakeholders in a participatory manner.

* they call stake holders from the Regional Referral Hospitals to come and input into the policy formulation or guidelines making [process] except that recently the SRH guideline we were not happy about the involvement because you don’t get a Consultant to revise a guideline which is going to affect the whole country no wonder it was rejected.****(KII_NLI009)***.

The diversity of the MCH cluster meant that they hold diverse forms of power such as knowledge, fiscal and political which is crucial in influencing the agenda and framing of the stillbirth issues in the country. Unique to the ongoing monitoring of policy implementation was the feedback loop linking subnational and national policymakers such as the Parliament of Uganda for up-to-date information on policy implications of the strategies implemented. Commenting on this relationship, a respondent observed;

*We have quarterly meetings with stakeholders at the districts and we share the data and now on the quarterly basis, we visit the parliament to brief it[them] on issues of maternal newborn to lobby for resources, lobby for attention which is a good platform.****(KII_NLI007)***

#### Policy entrepreneurs

Policy entrepreneurs have been critical in the national MCH agenda particularly newborn survival contributing through the MCH cluster and global collaborators on research and actively involved in global stillbirth working groups. The professional bodies particularly the Association of Obstetricians and Gynecologists of Uganda (AOGU) and Uganda pediatrics Association (UPA) were part of the strategic partnerships offering technical support to project implementation and policy. An Assistant Commissioner within the child health division of the Ministry of Health was designated as the national focal point officer for newborns to track country progress towards ENAP targets including stillbirth reduction[44]. The leader of the Centre of Excellence for Maternal and Newborn Health Research at Makerere University is a newborn health researcher who spearheaded the first Maternal and Newborn Health Conference in Uganda in 2015. As part of global stillbirth coalitions, he is involved in both global and national level advocacy and contribution to setting newborn research priorities [45]. Recognizing this contribution, a respondent thus noted;

*Maybe I start from the newborn committee at the Ministry and the School of Public Health; ……. doing his things in project mode but at least communicates and passes on the information and the evidence to the Ministry hoping that they would catch fire and continue.****(KII_NLI007)***

#### Credible indicators

National level stillbirth indicators were for long masked within perinatal mortality in the routine data. The sharpened plan relied on data from the Uganda Demographic and Health Survey (UDHS) 2011 to make a case for the national stillbirth burden. However, it was also reported under the perinatal mortality data to reflect the hidden burden. At the household, community, and facility level, the burden and effects of stillbirth were a felt problem. The lack of reliable stillbirth data triggered national efforts to address the issue. In response, the Ministry of Health first migrated to the DHIS2 in 2012/13 and by 2015, the country had a stillbirth rate of 21 per 1,000 total births[46]. It was later followed up with the inclusion of stillbirth as a notifiable condition captured through the surveillance systems and also as an indicator for monitoring district and health facility performance captured through the routine data systems. The focus was on responding to facility-based fresh stillbirths while interventions during antenatal care continued to address macerated stillbirth. Commenting on this approach, a respondent noted that whereas attention was paid to both, the focus was more on fresh stillbirths;

*They capture data for both macerated and FSBs but you know a fresh stillbirth can be easily more avoidable than an MSB. The factors are there like a mother comes and then you delay to operate, so all those things and they can be easily addressed. Of course for macerated stillbirths, we need to improve our quality of antenatal care which also still bits; much as mothers attend antenatal you may find that our antenatal care is still not quality.****(KII_NLI016)***

Commenting on the desire to improve quality of care as the rationale for prioritizing fresh stillbirths, another respondent thus noted;

*we are saying that the 3rd delay is dominating and If all the facilities are providing quality, we will be able to provide safe obstetric care so that we reduce on the fresh stillbirths.****(KII_NLI008)***

It was again echoed that the need to find the cause and identify possible interventions to address the problem was another reason for prioritizing fresh stillbirths;

*Because fresh is easy to prevent and you know fresh it has just died. So you want to quickly know what is it that has caused this baby to die, and how can we address this gap which caused the baby to die. The macerated is in antenatal, the woman is at home and you know that is a bit (.) these [fresh births] are easier to address than the macerated. (****KII_NLI009)***.

**Fig. 1:**
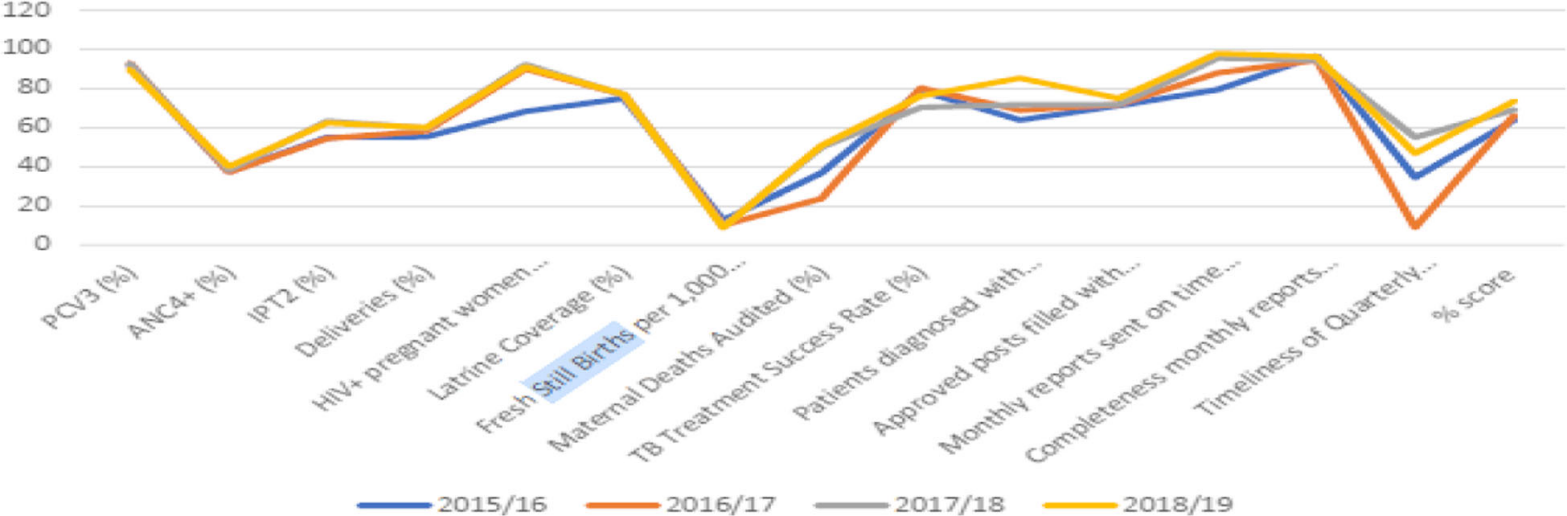
Source: Ministry of Health. Annual Health Sector Performance Report 2018/19

The 3rd Health Sector Development Plan (HSDP) translated both the ENAP and the UN Global strategy Every Woman Every Child into national policies[14] where facility-based fresh stillbirth reduction target as a health sector performance indicator was set at 11/1000 by 2020 using the 2013 baseline prevalence of 16/1000. This was in line with the ENAP national stillbirth reduction target of 12 or less by 2030 and 10 or less by 2035 if global stillbirth reduction targets were to be achieved[42]. Ever since performance has exceeded target year on and by 2018/19 the rate stood at 9/1000 above the HSDP target of 12/1000[17] with stillbirth consistently performing ahead of other indicators used for computing the district annual health performance (Fig. 1).

From 2016 stillbirths were included among the indicators for measuring and comparing health performance across districts. At the health facility level, it is a measure of the quality of care during antenatal care for macerated stillbirths and delivery services for fresh stillbirths[17]. For the national referral hospital, it is considered an indicator for measuring efficiency as viewed from inputs against outputs. Commenting on the role of documentation about facilitating reflections to devise strategies for improvement, a respondent thus noted;

*and then, of course, the issue of documentation, the HMIS but also locally be able to look at their own data in the Districts and identify what the problem is and of course the way they are working towards to locally address it…… I have been looking at stillbirths I would say that one of the things which came through with HMIS which was actually very important that we collect proper data right from the grass roots to the Ministry where it is analyzed and that actually shows where the problem is and once you have the problem then they identify what should be done to actually prevent the problem you see at the end.****(KII_NLI017)***

#### Focusing events

At the global level, the GAPPS conference in Seattle with major funders present was one of such focusing events to draw attention towards addressing stillbirths and come up with a roadmap[32]. The launch of the Global strategy Every Woman Every Child and the subsequent inclusion of stillbirths in the countdown reports [33] were the other focusing events with the turning point being the publication of the 2011 stillbirth Lancet series: call to action[2]. Another stillbirth Lancet series published in 2016 ending preventable stillbirth was also a key focusing event[22]. It drew attention to the potential of available low-cost interventions to address stillbirth risk factors[4]. The launch of the ENAP and its adoption during the World Health Assembly the same year drew political commitment from 194 member countries to address the problem. Consequently, some of the key targets and elements from ENAP were reflected in national guidelines such as the Health Sector Development Plan and the Sharpened Plan. A respondent thus noted;

*one of the big ones is the Sharpened plan. Having written this Sharpened plan, the next level will be the implementation. The implementation of the sharpened plan is actually working through the investment case. So investment case I think is an important area that is trying to translate the policy.****(KII_NLI004)***

The first Maternal and Newborn Health conference held in 2015 with support from Save the Children had an objective of linking the country’s ENAP outcomes, global research, and advocacy into action to support the implementation of national policies and guidelines was another such focusing event. The conference highlighted the national stillbirth burden and drew the attention of stakeholders towards doing things differently in the post MDG[41]. Other funding for newborn health interventions provided evidence for the policy as well as systems strengthening to address stillbirth. Among these was the World Bank’s launch of the Business plan for maternal and child health in 2015 is yet another focusing event for political prioritization of stillbirth in Uganda[40]. Its implementation in the country has seen fast-tracking of interventions at the subnational level which will see an implementation of activities like perinatal death audits and improving civic registration systems among others. A chronology of key events at the national level are presented in Table 3 below;

**Table 3:** Key events for national stillbirth prioritization

Year	Key event	Link to theme from global campaign	Objective and relevance to stillbirth response
2012	Roll out of the Saving Mothers Giving Life (SMGL) pilot project in Uganda	Integrated prevention. Evidence of cost effective interventions. Progress monitoring Actions against stigma	• Uganda was one of the two countries where intervention was rolled out with stillbirth reduction indicators • Piloting of evidence based interventions at district health systems level.
2013	Sharpened Plan 2013–2017	Country targets for stillbirth reduction. Integrated prevention Progress monitoring	• Reflection of stillbirth as indicator for subnational level outcome performance indicators • Stillbirth as an outcome indicator for tracking for a national reduction target of 11/1000 by 2020)
2015	ENAP country progress report with stillbirth as one of the indicator.	Progress monitoring	Tracking intervention effects on reducing stillbirth burden while monitoring progress.
2015	HSDP includes stillbirth as an outcome indicator for tracking with a national target of 11/1000 by 2020	Country target for stillbirth reduction. Establish burden and disparities	National commitment towards stillbirth reduction Improved quality of care around the time of delivery to avert most stillbirths happening at that time due too poor quality of service.
2015	Health Financing Strategy (2015/16-2024/25	Evidence of cost effective interventions. Integrated prevention.	Address financing bottlenecks to improve funding for health and align with international norm and funding opportunities. Alter incentive structure in health system to improve motivation at final point of service delivery and access to quality healthcare
2016	Investment Case – 2016.	Integrated prevention. Evidence of cost effective interventions.	Align with global funding opportunities Revised in the context to support implementation of key interventions addressing stillbirth risks
2016	AHSPR with stillbirths reflected as an indicator for district performance	Progress monitoring. Improve quality of care Establish burden and disparities.	Get stillbirth out of the shadow within the health systems by reporting the burden at subnational level. Responding to stillbirths occurring around the time of delivery as a reflection of the poor quality of care provided during late term and labour
2016	RBF framework institutionalization	Integrated prevention Evidence for cost effective interventions	Compensation for outputs including interventions to investigate and address stillbirth causes such as MPDRS among other maternal and child health prioritized interventions to incentivize performance at final point of service delivery.
2017	Revised MPDSR guidelines	Integrated prevention. Evidence for cost effective interventions.	Improved investigation of stillbirth cause and classification of stillbirth. BABIES matrix to guide review perinatal deaths incorporated from the SMGL project

#### Clear policy alternatives

Addressing stillbirths through attention to health systems strengthening coincided with government efforts towards the same in response to maternal and child mortality reduction. Global strategies highlighted in the 2011 Lancet Stillbirth series[7, 9] and the Every Newborn Action Plan 2014[8, 42, 47] observed the need for health systems strengthening through improved quality of services during delivery through Basic and Comprehensive Emergency Obstetric care due to its highest effect on stillbirths[10]. National strategies for achieving the MDG targets witnessed some interventions rolled out to improve emergency obstetric care services at the subnational level. HCIVs were to be headed by Medical Doctors specifically to deliver emergency obstetric care among other services[48], prioritization of training, and recruitment of rare cadres at HCIV such as anesthesiologists. Other initiatives included increased training and deployment of midwives at HCII to offer outpatient maternal health services and improving HCIII to provide inpatient maternal health services and Basic Emergency Obstetric Care among others[48].

The MoH Health systems strengthening plans were accelerated by the new PEPFAR change of strategy to scale down direct donor support announced by the US government in 2012 [49]. PEPFAR the lead financer for HIV response was switching from emergency response to targeted sustainable approach with greater country ownership [50] under the PEPFAR 3.0 (2013–2019) strategy aiming to maximize evidence-based intervention through the impact of investment by providing technical support. Part of the transition process included the targeting of resources to high burden regions through geographical pivoting [51] where facilities were prepared to receive that support from the government. During 2012/2013 FY the government spent 7.4 % of the annual budget towards financing health systems strengthening [52]. These would later turn out to be the same interventions to improve maternal health services thereby prevent stillbirth, especially at the facility level. The emphasis for improved care during ANC received attention right from the MDG era with interventions for early reporting for the first antenatal care visit and increase completion rates of the recommended four visits. Interventions like focused antenatal care (FANC), male involvement in birth preparedness, addressing the distance to health facilities, provision of MAMA kits for pregnant mothers, the Village Health Team strategy. Although global stillbirth campaigns recommended improvement in the delivery of advanced antenatal care services, they cautioned that it would come at a higher cost, and yet the call was for the implementation of interventions that suited the health systems capacity to deliver the same. This resonated well with another recommendation calling for delivery of linked services.

### National political environment

#### Political transition

A major political transition that shaped the acceptance and integration of global stillbirth recommendations into national priorities was the decentralization system of service delivery[48, 53]. Under the arrangement, decision-making responsibilities were delegated at the subnational level with a focal person in charge of maternal and child health services at every district. Although these processes had happened sometime back, they worked to anchor interventions responding to stillbirth at the subnational level. Unique to the health sector, another managerial layer below the district and headquartered at HCIV known as the Health Sub-district introduced to improve management at the subnational level[53]. Infrastructural improvements have seen renovations and upgrading of functional maternity wards and operational theatres to provide Comprehensive Emergency Obstetric Care (CEmONIC) while providing mentorship and supervision for lower-level facilities. It is from this structure that national efforts to operationalize the global campaign strategy of improving access to quality maternal health services are being delivered.

Health in Uganda particularly the poor state of maternal and child health services has been a sensitive political issue attracting attention during political sentiments which have previously led to the scrapping of user fees and salary enhancement for health workers[53]. The national commitment to improving maternal health services stems from being a signatory to global agreements about improving maternal health such as the MDGs and Sustainable development Goals [54, 55]. Reproductive health services are highlighted as part of the tracer indicator for monitoring the country’s progress towards Universal Health Coverage (UHC) targets[56]. The country’s engagement of the private sector improved coverage of MCH service delivery through the private sector and was operationalized when Uganda rolled out the health financing strategy (2016/2025) which introduced reforms in pursuit of health sector progress towards UHC. It laid the foundation for Results-Based Financing the main financing mechanism for the GFF with a strong component of reimbursing facilities for outputs attained including perinatal death reviews[40]. This project has a strong component of interventions responding to stillbirth.

As a result, maternal health services face a web of interlocking accountability mechanisms comprising of the political, administrative, and technical. Within the district league table, SB was included as a performance indicator from 2016/17 meaning that leaders charged with accountability at that level have to monitor to ensure stillbirths don’t happen to improve district performance. Civil society organizations all exert accountability to ensure the delivery of quality maternal health services in the country. Previously Civil Society Organizations (CSO’s) have successfully lobbied Parliament to block the health budget if it didn’t address systemic health systems challenges leading to a reallocation of approximately $15 million to address health worker shortage. They have also used strategic litigation as a political tool to influence norms and steer processes towards social change aimed at pushing the government to be more accountable to maternal deaths[57].

#### Competing health priorities

Maternal and child health enjoys political attention due to the sensitivity of the indicators associated with it and the momentum built during the MDG era. Neonatal survival received global attention that trickled into national interventions. Grants supporting projects with neonatal components increased and this further catapulted neonatal survival within the donor community. The health sector’s pursuit of health systems strengthening strategy to build capacity has seen HCIVs equipped with functional theatres, recruitment of anesthesiologists, and laboratory technicians to support the delivery of CEmONIC. Elsewhere midwifery skills have been strengthened to deliver PMTCT and maternal health services and health facility data improvement through Continuous Quality Improvement. The outcome of which has seen innovative strategies such as integration of services within the Reproductive Maternal Newborn and Child Health (RMNCAH) continuum of care making maternal health one of the highly prioritized RMNCAH indicators. The district scorecard has stillbirth as one of the outcome indicators (Sharpened plan) while district performance in health is assessed based on selected indicators including stillbirth[17].

## Discussion

This study sought to establish reasons and provide explanations that led to the rise of political priority for stillbirths in Uganda. The framework factors support providing explanations for the observed developments with regards to prioritizing stillbirths. Using the applied framework, results show important aspects that may have played a role in prioritizing stillbirth reduction. Despite its neglect, the global burden and feasibility of implementing available proven low-cost interventions with the highest impact merited its attention. It also received the benefit from its linkage with maternal and newborn mortality risk factors and the potential of interventions to address all. Being driven by an established and powerful policy community already converged around neonatal survival who was at the time working on the Lancet neonatal survival series 2003.

Attention to stillbirths appears to have built momentum as an offshoot of earlier newborn survival global campaigns that sought to correct the prevailing assumption then that newborn health was automatically being addressed through existing maternal and child health programs [21]. In this context, subsequent national-level efforts can be traced to the consistent and protracted global campaigns around maternal and neonatal mortality reduction which later culminated into newborn survival campaigns. The earliest recognition of stillbirths as a global problem is traced from its omission as one of the indicators for tracking progress towards the attainment of MDG targets. The team compiling the countdown to the MDG report was already an established policy community that was working on the Lancet newborn survival series. This may have worked to bring the issue close to what the policy communities were already working on, hence the reflections of stillbirths in the 2010 countdown report[12]. Despite its strong grounding in MCH, stillbirth still receives slightly less attention at the national level compared to maternal and child mortality reduction.

In this study, the international norm promotion is seen to have influenced the national level prioritization of stillbirth. This was key as it triggered the other factors like availing of funding for interventions with stillbirth reduction targets and focusing events. Consistent with what has been revealed elsewhere [21] the motivation to act in response to reducing stillbirth shares aspects from what was highlighted at the global level. Shiffman contends that other than the material factors such as data on the national stillbirth burden, the power of ideas, and how they are framed may draw more attention to the problem. At the time, indications were that more countries were likely to miss out on MDG 4 because the rate of mortality reduction in neonates was slower compared to children over 28 days hence the focus on neonatal survival was extended to stillbirth reduction. In Uganda, stillbirth data was being captured primarily using facility-based records at that time (AHSPR 2011/12). Many of the community cases were going unrecognized due to negative cultural practices of secretive burial practices and the stigma it caused to the bereaving family[16]. Similar findings have been reported from Bangladesh, Malawi, and Bolivia where international norm-setting for the reduction of newborn deaths opened final windows of opportunity for national governments to act on the problem[19, 58].

The spark to policy formulation and intervention rollout to address stillbirths is attributed to resource provision particularly financial and technical support from global stakeholders. Increased funding for newborn health in Uganda played a role in varying ways. Consistent with political prioritization literature, the alignment of policy to potential funding led to the rollout of the Investment case which highlighted some interventions to address stillbirth in Uganda in time for the World Bank funding [59]. Similarly, the revision of the MPDSR guidelines with a strong component on auditing perinatal deaths was informed by the evidence from supported projects reflecting the feasibility of some of the tools recommended in the guidelines[39]. Further, recommendations for improved stillbirths reporting emerged from collaborative participation in evidence generation with global actors [60] similar to what has been reported elsewhere[58].

A cohesive policy community as articulated by Shiffman [20] in the framework was key in influencing the prioritization of stillbirths. Already these were familiar and working on the national maternal and child health agenda. The MCH cluster was highlighted as having been swift in adopting global recommendations into the national context to inform policy and practice. Provision of evidence into policy and translation of the ENAP strategies and targets into the Health Sector Development Plan[14] and the Investment case[59] are some of the examples. A number of the political entrepreneurs were already advocates for the MCH and not exclusive to stillbirth. This background helped generate ground from which stillbirth prioritization was anchored. None of the political entrepreneurs championed stillbirths in isolation of maternal and child health. The fact that the framers of stillbirth as a major public health problem pointed to the feasibility of implementing recommended strategies along the continuum of care and in an integrated manner meant that the policy community to advance it would also have interest in maternal and child health with a promise of a triple return on investment from such interventions.

When targets were set for stillbirth reduction[14], the Ministry of Health had specifically focused on addressing those risk factors occurring intrapartum. Integrating these indicators within ongoing quality improvement efforts at health facilities meant that while working on achieving the targets the general quality of maternal and child health services was also improving. Inclusion of stillbirth into the district league table meant that those charged with accountability would ensure this indicator performs well to raise district ranking among peers. This could partly explain why stillbirth performed well compared to other indicators in the duration following the rollout of the Health Sector Development Plan. Experience from implementers shows that whereas perinatal reviews should be conducted on all cases, they were mainly conducted for early neonatal deaths [17] calling for targeted follow-up beyond policy provision.

The framework emphasizes the importance of focusing events in drawing attention to the public health problem. Our results indicate that some focusing events such as integration of ENAP targets into national policies, the first maternal and neonatal health conference, and funding triggered the acceleration of stillbirth prioritization. Related to the policy alternatives, the focus of implementing strategies along the continuum of care in an integrated manner resonated well with the Ministry of Health’s aspirations at the time in addition to health systems strengthening.

Other than the political transition as proposed from the framework, the decentralization of health services had happened some time back and what was put in place as a result only helped to anchor the recommendations to address stillbirth at the subnational level in Uganda. On the contrary, the decentralization process in Indonesia is reported to have hurt the prioritization of safe motherhood[20]. The time of accelerated advocacy to prioritize interventions addressing stillbirth coincided with the post-MDG health agenda driven in part by the desire to attain Universal Health Coverage[14]. This finding compares well with what has been established elsewhere for having facilitated prioritization of maternal and newborn survival[21]. The sensitivity of maternal and child health as a political issue benefited the prioritization of interventions to address stillbirth[48]. Already the country was implementing interventions to address persistent MCH problems many of which have the potential to address stillbirth.

The role of civil society in raising attention to maternal and child health has been observed elsewhere[57]. Consistent with this observation, civil society played a key role in raising the maternal health issue especially the poor quality of emergency obstetric services. They engaged in strategic litigation aimed at the compelling government to improve the quality of maternal health services through proposed interventions with the potential to address stillbirth risk factors. Consistent with what has been reported elsewhere [58] we did not find competing priorities that may have impeded stillbirth prioritization but that favorable factors that helped it accelerate. Maternal and child health programs were already being prioritized by the Ministry of Health and had been earmarked as one of the indicators for tracking national progress towards Universal Health Coverage[56].

## Limitations

This study had some limitations; first, the study analyzed events retrospectively and where no proper documentation of events existed, results would be subject to recall bias of the respondents. The applied framework also had limitations whereby some of the factors within the framework were contextually not applicable. A case in point is the political transition which in our case the decentralization of health services had happened sometime back and the established structures only worked to anchor recommended interventions from the global campaign but not because they were intended for that in the first place.

## Conclusions

The application of the framework helped unveil the factors behind the prioritization of stillbirth reduction in Uganda. The transnational influence played a key role in triggering interest in the issue during the initial stages of raising attention to the problem. The success and subsequent processes however relied so much on domestic advocacy and the national political environment. The key factors for this include the cohesive policy community converging around maternal and child health which was able to embrace the recommendations from the global campaigns into policy priorities. Political transition, in this case, appealed more to systems improvement through proposing new policies and guidelines to streamline implementation of interventions which was an important factor in addition to the health systems capacity to embrace the recommendations.

From our analysis, it appears that political transition is more applicable in settings with major political events. From our study, we observed that small-scale incremental changes that later played a role in facilitating prioritization of stillbirth reduction also happened during the processes. Mahar and Sridhar 2012 summed it as the role of institutions in the priority generation process [61]. Within the Ugandan context, the establishment of the MCH cluster within MoH in a way accelerated the political prioritization of stillbirth reduction. Indeed, the principal architect of this framework acknowledges this shortcoming in understanding the political prioritization for neonatal mortality reduction in Bangladesh [58]. He notes that political transition is applicable when its meaning is stretched. We observe from our case study that in the absence of fundamental political events in the country, incremental institutional transformations occurred and which were crucial in shaping political prioritization of stillbirth reduction. At the global level, McDougall (2016) notes that the expanding power of non-state actors in global health governance space in a way has influenced political prioritization[62]. Globally private philanthropies are increasingly getting immersed in policy communities that shape political prioritization. Similar occurring extends to national-level efforts. We have argued earlier that the policy community converging around the MCH cluster within MoH with representation from diverse stakeholders worked to synthesis the evidence which later informed policy provisions and prioritization of stillbirth reduction. Future application of this framework would benefit more from incorporating peculiar aspects like institutional/organizational transformation onto the political transition factors. To this end, we propose a revision or modification to include organizational/institutional transformations to cater for the small-scale incremental changes to the institutions and health systems that are targeted for political prioritization of global health issues.

## Supplementary Information

**Additional file 1:**

## Data Availability

The datasets generated and/or analyzed during the current study are not publicly available due to ethical obligations to keep respondents’ confidentiality but are available from the corresponding author on reasonable request.

## Abbreviations

ANC: Antenatal Care
AOGU: Association of Obstetricians and Gynecologists of Uganda
APHSR: Annual Health Sector Performance Report
CEmONIC: Comprehensive Emergency Obstetric Care
CSO’s: Civil Society Organizations
ENAP: Every Newborn Action Plan
GAPPS: Global Alliance to Prevent Prematurity and Stillbirth
HC: Health Centre
HSDP: Health Sector Development Plan
ISA: International Stillbirth Alliance
LMIC: Low and Middle Income Country
MCH: Maternal and Child Health
MCH-TWG: Maternal and Child Health-Technical Working Group
MDG: Millennium Development Goals
MoH: Ministry of Health
MPDSR: Maternal and Perinatal Death Surveillance and Responses
NGOs: Non-Governmental Organizations
RMNCAH: Reproductive Maternal Newborn and Child Health
UDHS: Uganda Demographic and Health Survey
UHC: Universal Health Coverage
UPA: Uganda Pediatrics Association
